# Development of the long COVID – 6 dimensions quality of life (LC-6D-QoL) scale: a Delphi study

**DOI:** 10.1186/s41687-026-01084-3

**Published:** 2026-05-20

**Authors:** Esther Ortega-Martin, Javier Alvarez-Galvez

**Affiliations:** 1 Department of General Economy (Health Sociology Area), Faculty of Nursing and Physiotherapy, University of Cadiz, 52, Av. Ana de Viya, Cadiz, 11009 Spain; 2 Computational Social Science DataLab, University Research Institute for Sustainable Social Development, University of Cádiz, Av. de la Universidad, Jerez de la Frontera, 11405 Spain

**Keywords:** Long COVID, Quality of life, Delphi study, Patient-reported outcome, Health measurement, Instrument development

## Abstract

**Background:**

Long COVID significantly impairs quality of life through multi-systemic complexity, including fatigue, cognitive dysfunction, and dysautonomia. Existing generic tools fail to capture these manifestations, limiting patient-centered assessment. To address this gap, this study proposed a patient-focused, 16-item scale developed and content-refined through expert consensus, to evaluate quality of life in people with Long COVID.

**Methods:**

A two-round Delphi study involved two rounds of questionnaires and feedback. 37 experts with clinical or research experience in Long COVID participated in the first round, and 26 completed the second. In the first round, experts rated the relevance of proposed indicators. The second questionnaire incorporated modifications based on the experts’ comments. Quantitative data were analyzed using descriptive statistics and agreement measures, while qualitative comments were thematically coded.

**Results:**

Experts reached substantial agreement, above 83%, on the dimensions covering the main aspects of quality of life. Based on participants’ comments, the initial scale was modified, and a consensus between 80 and 92% was reached in all dimensions. The Delphi process resulted in a scale composed of 6 dimensions and 16 items, which include dysautonomia assessment and affective-sexual life impact, omitted in generic tools. The dimensions of the LC-6D-QoL Scale were: (1) general health; (2) physical health; (3) mental health; (4) daily functioning; (5) social relationships and support; and (6) economic and work-related aspects.

**Conclusions:**

This study establishes an expert-informed foundation for future validation of the LC-6D-QoL Scale, integrating clinical and experiential knowledge to refine its preliminary structure. The LC-6D-QoL Scale is a patient-centered instrument capturing six key dimensions often missed in generic tools—such as dysautonomia, mental fatigue, and work-related impact—to better assess quality of life in Long COVID.

**Supplementary Information:**

The online version contains supplementary material available at 10.1186/s41687-026-01084-3.

## Introduction

Long COVID (LC) is an emerging chronic condition that affects 6–15% [1–5] of people following SARS-CoV-2 infection, significantly impacting their quality of life (QoL) [6, 7]. LC affects multiple organ systems and manifests in a wide variety of symptoms, the most prevalent being fatigue, cognitive impairment, post-exertional malaise, psychological symptoms (e.g., anxiety, depression and PTSD), shortness of breath, and pain [2, 8–13]. This clinical heterogeneity necessitates QoL instruments capable of capturing its unique multisystemic complexity [14, 15].

In this context, a major challenge is the lack of specific, sensitive and validated tools to adequately evaluate its effect on patients’ QoL. Although generic instruments are frequently used—74 PROMs have been identified in LC research, with the EQ-5D-5 L being the most common [16]—these tools were not designed to capture LC’s manifestations or the nuances of patients’ lived experience, and they often fail to represent the condition’s heterogeneity. This measurement gap is critical, as the systemic burden of LC remains understudied [17]. Consequently, a patient-centered assessment tool becomes indispensable for both clinical care and health system planning.

To overcome limitations of generic tools, disease-specific instruments like the PAC-19QoL [18] represent a significant advance by identifying Quality of Life Indicators (QoLIs) relevant to LC. While this scale integrates psychological, physical, social, and occupational domains, critical gaps persist. Emerging evidence reveals persistent challenges in clinical management—particularly regarding dysautonomia [19, 20], and psychosocial challenges like social misunderstanding, loss of autonomy, and occupational vulnerability [21, 22]. Importantly, qualitative data from a previous study with LC patients [21] were used to ensure that the conceptual foundation of this new instrument is patient-centered and reflects real-world experiences, highlighting the central role of the patient voice in its development.

The prior qualitative study [21] involved semi‑structured interviews with 23 patients with LC in Spain, analyzed using thematic analysis [23] with double coding and saturation. While that exploratory study did not include cognitive debriefing, such evaluation was conducted during the Delphi process: experts (including patients) evaluated the clarity, relevance, and comprehensibility of each item, leading to refinements documented in the changes made between rounds. This approach was informed by COSMIN guidance for content validity and PROM development [24].

The primary qualitative study was prioritized for two reasons. First, the lived experience of LC is recent, rapidly evolving, and context‑dependent; published qualitative evidence from Spain was limited at the time, and generic literature may not capture country‑specific healthcare barriers or cultural nuances. Second, direct patient elicitation ensures that the instrument reflects current, context‑grounded perspectives. The intended context of use for the LC-6D-QoL is clinical practice and research, for assessing and monitoring health‑related quality of life in individuals with LC.

Given the limited and still evolving empirical evidence on LC, the Delphi method is particularly appropriate, as it has been widely recognized for its usefulness in contexts where empirical evidence is limited or scattered [25–27]. Therefore, this technique has been applied in LC research to establish diagnostic definitions in adults [28, 29] and in the pediatric population [30, 31], as well as to establish clinical criteria for identification and management [32, 33].

This research contributes to addressing this need by developing a patient-centered, contextually sensitive instrument to assess QoL in individuals with LC. The Delphi methodology was used to refine the instrument’s content through expert consensus, integrating patient-derived concepts identified in previous qualitative work with expert consensus. This approach ensures that the scale reflects both the lived experiences of patients and the clinical knowledge of experts.

To address these limitations, we proposed the Long COVID – 6 Dimensions Quality of Life (LC-6D-QoL) Scale, a patient-centered instrument designed to comprehensively evaluate QoL in LC. Using Delphi methodology, we aimed to:

1. Establish the expert-informed foundation for future validation of the scale, through consensus on the relevance and clarity of the proposed domains and indicators.
2. Integrate patient-derived insights to reflect lived experiences.
3. Develop a multidimensional tool encompassing physical, mental, social, occupational, and economic impacts.

## Methods

### Study design

To develop the content of a QoL scale tailored to individuals with Long COVID (LC), the LC-6D-QoL Scale, a Delphi methodology was employed. This structured, iterative process engaged a multidisciplinary panel of experts to evaluate and refine proposed dimensions and indicators through successive rounds of questionnaires and feedback, aiming to achieve consensus on the most relevant and meaningful components of the instrument. The study protocol was not prospectively registered, as this was a Delphi consensus study rather than a clinical trial.

### Participants

Given the emergent nature of LC, our selection criteria prioritized current clinical engagement and specialized knowledge over years of experience. We assembled a panel of 37 experts through a purposive sampling strategy that deliberately sought to include the diverse range of professionals involved in LC care. The sample size (37 experts in round 1, 26 in round 2) was based on Delphi methodological guidance, which recommends 15–30 participants per panel to achieve stable consensus while allowing for attrition [34]. We actively recruited from two primary sources: (1) the Spanish Research Network on Long COVID (REiCOP) and (2) the research team’s professional networks, which we used to identify and invite experts from underrepresented specialties to ensure a comprehensive perspective. Participants were selected based on their active involvement in LC clinical management or research, with invitations distributed via email containing a link to a self-administered online questionnaire (Google Forms).

Inclusion criteria required:

- Clinical experience: Direct patient care for individuals with LC within the past 24 months, or
- Research experience: Active involvement in LC-related research, evidenced by authorship in peer-reviewed publications on LC or participation in ongoing LC studies within the past 24 months.
- Current engagement: Ongoing involvement in LC patient management or research at the time of recruitment.
- Commitment to participate in all Delphi rounds.

Exclusion criteria:

- No LC-related clinical or research activity within the past 24 months.
- Inability to commit to the full Delphi process.

For the four experts who self‑identified as patients, additional inclusion criteria were: self‑reported diagnosis of LC (meeting WHO definition), age ≥ 18 years, and fluency in Spanish. No additional exclusion criteria were applied.

All selected participants met these criteria, ensuring the panel represented practical, frontline expertise in LC care. We chose to prioritize this hands-on experience over rigid demographic quotas, so that the scale’s content would genuinely reflect real clinical needs. This approach was particularly appropriate given LC’s recent emergence, as it allowed us to capture contemporary clinical insights without arbitrary experience thresholds that might exclude valuable perspectives. The perspectives of people with LC were incorporated in two ways: First, the initial set of dimensions and indicators used in the Delphi rounds was informed by a prior qualitative study [21], which explored the lived experiences of individuals with LC. Second, four members of the expert panel also self-identified as patients, offering a dual perspective that enriched the consensus process. In this study, the term “experts” refers to all panel members, including patients, clinicians, and researchers. Patients participated in both Delphi rounds, rating the relevance of indicators and providing qualitative feedback on clarity and comprehensibility. Their role was identical to that of other panel members.

No financial compensation was provided to any participant. No formal steering committee was established. The research team (including a patient co‑investigator) designed the study and analysed the data but did not participate as members of the Delphi panel.

### Data collection

For the first round, an initial questionnaire was designed that included a set of proposed indicators for assessing QoL in LC. Experts were asked to rate the relevance and appropriateness of each indicator using a Likert scale (ranging from 1 = ‘not relevant at all’, to 5 = ‘very relevant’), and to include open-ended comments for suggestions for new indicators, adjustments or improvements in the formulation of existing ones. Item clarity and comprehensibility were evaluated during the first Delphi round through qualitative comments from all panelists.

Dysautonomia was included as a single item within the Physical Health dimension. This placement was based on patient qualitative data, which described symptoms such as dizziness, palpitations, and sweating upon standing as integral to the physical burden of LC. Future factor analysis may test whether these items warrant a separate dimension.

All items use a recall period of “the last month”. This balances the high symptom variability in LC against patient recall burden, following general PROM guidance that recall periods should consider both the nature of the condition and patient burden [35].

Based on the analysis of the responses from the first round, including quantitative assessments and qualitative feedback, a second revised questionnaire was developed. This second questionnaire incorporated modifications to the indicators and questions based on the experts’ comments. The experts were presented with a summary of the group responses from the first round (presented anonymously) and asked to re-evaluate the indicators, providing additional comments. They were asked to reconsider their assessments in the light of the group’s overall evaluation and to prioritize those indicators considered most relevant.

The first Delphi round started on 19 December 2024 and remained open for three weeks to accommodate the winter/Christmas holiday period. The second round took place from 12 February 2025 and remained open for two weeks. All participants were informed in advance of the time and received reminders to encourage them to complete the survey by the deadline.

### Data analysis

Quantitative data from both rounds were analyzed using descriptive statistics and measures of agreement. Table 1 provides a summary of the data analysis strategies used in the study.

**Table 1:** Summary of data analysis strategies used in the study

Data analysis	Description
Descriptive statistics	Means, standard deviations (SD^a^) and frequency distributions were calculated for each item. Levels of agreement were categorized as: high (≥75% of scores of 4 or 5), moderate (60%-74%) and low (<60%), aligning with consensus thresholds where 75% represents the observed median and is the most common Delphi consensus cut-off [34]. This pre-defined threshold of ≥75% agreement served as the stopping criterion for the Delphi process, which was therefore concluded after the second round.
Agreement analysis	The adjusted Kendall’s coefficient of agreement (Wt^b^) was used to assess the degree of agreement between experts.
Variability of responses	Standard deviations per dimension were analyzed as indicators of response dispersion. In line with the literature, reductions in SD between rounds were interpreted as a sign of increasing consensus and response stability [34].
Qualitative analysis	Open-ended comments were thematically coded by two researchers through iterative review, identifying patterns and proposals for item improvement. Intercoder reliability was ensured through comparative coding, with discrepancies resolved by consensus. Consensus-based decisions were subsequently made on wording clarifications and structural adjustments.
Prioritization and consistency	Kendall’s W coefficient was used to assess the degree of consensus in the ranking of indicators between both rounds, complemented by the interquartile range (IQR^c^) to analyze the dispersion of responses.

Legend: ^a^SD: standard deviation; ^b^Wt: adjusted Kendall’s coefficient of agreement; ^c^IQR: interquartile range. Descriptive statistics set the stopping rule (≥75% consensus); Kendall’s W and IQR assessed agreement and prioritization; SD tracked convergence; qualitative analysis guided item revisions

For the interpretation of Kendall’s W, we followed Schmidt’s guidelines. Values below 0.5 indicate weak agreement, values between 0.5 and 0.7 indicate moderate agreement, and values above 0.7 indicate strong agreement [36].

All analyses were performed in the R environment (version 4.2.2), developed by Posit Software, PBC [37].

### Ethics approval

This study was performed in line with the principles of the Declaration of Helsinki. Approval was granted by the Ethics Committee of Cadiz (PEIBA 0659-N-23). All participants were previously informed about the objectives of the study and provided their informed consent by agreeing to participate through the online questionnaire platform. In addition, anonymity and confidentiality were ensured throughout all phases of the study, particularly among participants, who had no access to the identity or individual responses of other panel members.

## Results

### Expert panel participation

37 experts participated in the first round and 26 in the second round (30% attrition), from a variety of disciplines. Among them were 18 medical professionals, including specialists in internal medicine (6), family and community medicine (6), pulmonology (2), preventive medicine and public health (2), psychiatry (1), and microbiology (1). The panel also included nurses (3), a nursing assistant (1), physiotherapists (3), psychologists/neuropsychologists (5), pharmacists (2), specialists in nutrition/bromatology (2), cognitive stimulation professionals (2), a sexologist (1), and a social worker (1). The length of experience with patients with Long COVID (LC) ranged from 1 to 5 years, with a significant representation of professionals who initiated care from the early phases of the pandemic (between April and June 2020). In terms of familiarity with QoL assessment tools, 91.89% of participants considered themselves very or moderately familiar, which ensured a solid basis for assessing the indicators proposed in the study. Geographically, the panel was composed primarily of experts from Spain, with representation from different regions across the country, and included one participant from Mexico. The four patient‑experts were also healthcare professionals. No systematic disagreements between their views and those of other panelists were observed regarding the inclusion of any dimension.

### Consensus by rounds

In the first round, most experts agreed that the dimensions covered key aspects of QoL (86.49%), that the questions were understandable (86.49%) and that the response scale was appropriate (83.78%). However, some dispersion was observed across items, with standard deviations ranging from 0.87 to 0.94. Kendall’s coefficient of agreement was *W* = 0.63 (*p* < 0.001), reflecting a substantial level of agreement for the ordering of the dimensions. In addition, the standard deviations observed across core items (0.87–0.94) suggested moderate variability, indicating that although consensus was high, expert ratings were still moderately dispersed at this stage.

**Fig. 1:**
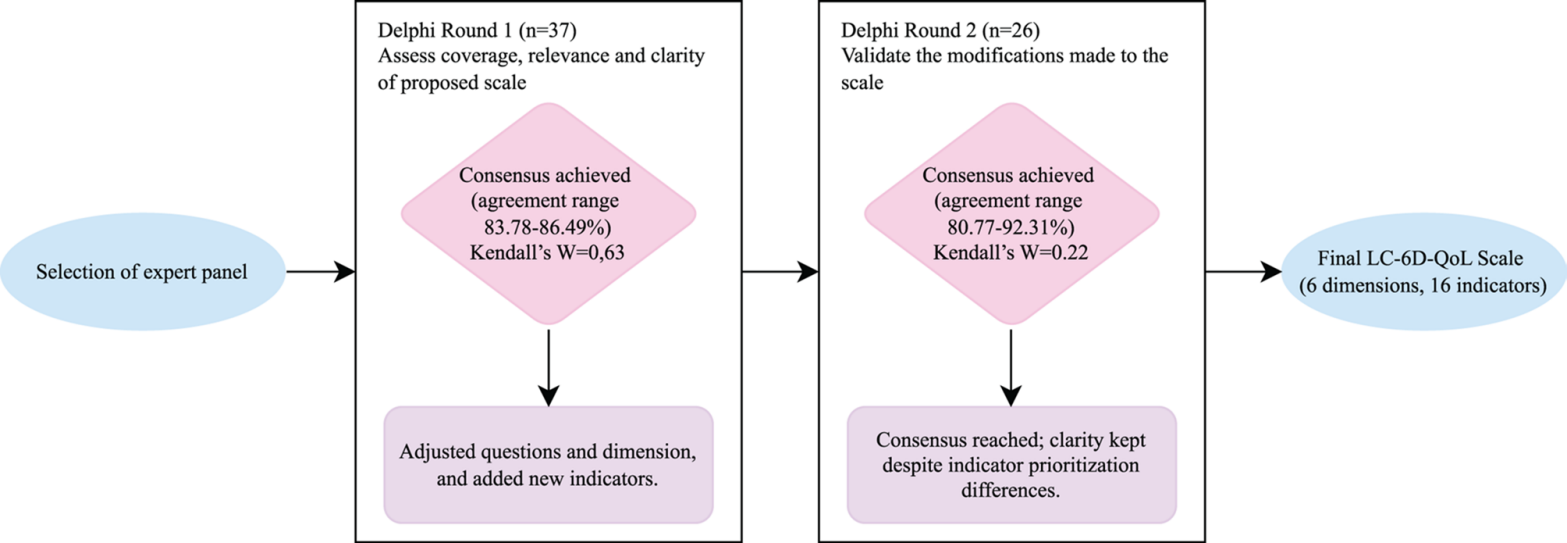
Delphi process flowchart showing consensus development and key methodological steps. Legend: consensus percentage and Kendall’s W values are shown for each round. Kendall’s W was significant in both rounds (*p* < 0.001)

Based on the qualitative comments, modifications were introduced and evaluated in the second round. In this phase, all dimensions reached a consensus above the pre-established threshold of 75%, ranging from 80.77% to 92.31% (more detailed information broken down by indicator in the supplementary information table). In particular, the dimensions with the highest consensus were dysautonomia and quality of social relationships (92.31%), followed by affective life and appropriate indicators (88.46%). Moreover, a decrease in variability was observed: the interquartile range (IQR) was 1.00 in all dimensions except ‘appropriate indicators’ (IQR = 0.00), indicating a greater homogeneity in the responses. Along with the consensus on the inclusion of indicators, the panel was asked to rank the indicators according to their relevance. Kendall’s coefficient for this prioritization was low (*W* = 0.22, *p* < 0.001), suggesting low agreement in the hierarchy assigned to the items. This decrease was also reflected in the standard deviations for the second round (ranging from 0.70 to 0.87), confirming a reduction in dispersion and a convergence in expert opinions. The evolution of consensus and agreement metrics across both Delphi rounds is summarized in the flowchart (see Fig. 1).

Moreover, there was a trend towards stability across rounds, as reflected by the decrease in measures of dispersion. Variances decreased between rounds 1 and 2, and the interquartile range remained low or zero for most attributes, indicating that participants’ ratings became more uniform as time progressed. The lack of notable changes in qualitative assessments in round 2 further reaffirmed the consistency of responses, validating that those involved had solidified their perspectives.

Given that the consensus was both high and consistent, and variability in ratings was low, the Delphi process was concluded at round 2.

### Changes made between rounds

The initial items were directly derived from the prior qualitative study, and following expert feedback, several adjustments were made and two new items were included. As a result of the first round, several relevant adjustments to the questionnaire were implemented (see Supplementary Information for all changes). Notably, all the modifications between rounds were based on specific participant comments. First, the dimension ‘Daily functioning and health perception’ was split into two separate dimensions: ‘Daily functioning’ and “General health”, concluding with a total of 6 dimensions following direct panelist recommendations. All dimensions were also reorganized, following the order suggested in the first round (see Table 2).

**Table 2:** The LC-6D-QoL scale following the consensus of the Delphi panel

Dimension	Indicator	Question	1	2	3	4	5
1.General Health	1.1. General health	How would you rate your **overall state of health**?	Very poor	Poor	Fair	Good	Very good
2. Physical Health	2.1. Pain	In the last month, how much **pain** has interfered with your daily life (including work and household tasks)?	Very much	Quite a lot	Somewhat	A little	Not at all
	2.2. Physical fatigue	In the last month, to what extent have you felt **physically fatigued** while performing everyday activities?	Very often	Quite often	Sometimes	Rarely	Never
	2.3. Functionality	In the last month, to what extent did you feel limited when making **moderate effort**, carrying groceries, climbing stairs, or walking for an hour?	Very much	Quite a lot	Somewhat	A little	Not at all
	2.4. Dysautonomia	In the last month, have you experienced symptoms such as **dizziness, rapid heartbeat, or excessive sweating** when changing position (e.g., standing)?	Very often	Quite often	Sometimes	Rarely	Never
3. Mental Health	3.1. Emotional	In the last month, to what extent have you felt **depressed or anxious**?	Very much	Quite a lot	Somewhat	A little	Not at all
	3.2. Mental fatigue	In the last month, to what extent have you felt **mentally fatigued** while performing everyday activities?	Very much	Quite a lot	Somewhat	A little	Not at all
	3.3. Cognitive problems	In the last month, have you had difficulties **concentrating or remembering** essential information (e.g., appointments, names, or tasks)?	Very much	Quite a lot	Somewhat	A little	Not at all
4. Daily Functioning	4.1. Limitations in daily activities	In the last month, to what extent have you felt **limited** in performing daily tasks, participating in leisure activities, or moving freely?	Very often	Quite often	Sometimes	Rarely	Never
	4.2. Personal autonomy	In the last month, how would you describe your level of **autonomy** in performing daily activities (personal hygiene, medication management, cooking)?	Very poor	Poor	Fair	Good	Very good
5. Social Relationships and Support	5.1. Quality of social relationships	In the last month, to what extent did you feel your family and friends **understand your situation and support** you?	Very much	Quite a lot	Somewhat	A little	Not at all
	5.2. Social isolation	In the last month, how often have you had **contact with friends**?	Never	Rarely	Sometimes	Quite often	Very often
	5.3. Affective and sexual life	In the last month, how much has your health affected your **romantic or sexual life**?	Very much	Quite a lot	Somewhat	A little	Not at all
6. Economic and Work-related Aspects	6.1. Loss of income	In the last month, how much has your health affected your **income**?	Very much	Quite a lot	Somewhat	A little	Not at all
	6.2. Work performance	In the last month, to what extent has your health affected your **work or academic performance**?	Very much	Quite a lot	Somewhat	A little	Not at all
	6.3. Return-to-work challenges	In the last month, have you needed **adaptations at work** (flexible hours, team support, or task adjustment)?	Very much	Quite a lot	Somewhat	A little	Not at all

A new indicator was added in the dimension ‘Physical Health’ related to dysautonomia, which was not covered before. In addition, a new indicator on emotional and sexual life was included in the dimension ‘Social Relationships and Support’, with the aim of better capturing the impact of the disease on life as a couple. This addition directly addressed panelist suggestions such as: “Within social relationships and support, I would include family relationships and within health perception … I would include sexual relationships”.

Several items were reformulated to improve clarity and appropriateness. The social support indicator was redefined to focus on the perception of understanding and support from the immediate environment, based on specific suggestions like: “I would change Social support perception to: to what extent did you feel your family and friends understand your situation and support you?”. The emotional health indicator was expanded to include both depressive and anxious symptoms, aligning it better with patients’ needs. In the work-related domain, the reference to ‘job instability’ was replaced by a more focused approach to job or academic performance, allowing for applicability to different profiles, prompted by insights such as: “It could include stagnation or impairment of work/academic activities …”.

### LC-6D-QoL scale structure

The final scale version included 6 dimensions and 16 indicators expressed as a single-item question with a 5-point Likert response format. Table 2 provides a complete list of indicators and response options. A full consensus breakdown per indicator can be found in Supplementary Information.

## Discussion

This study aimed to develop a specific patient-centered instrument to assess QoL in people with Long COVID (LC), addressing the current paucity of instruments tailored to the unique characteristics of this disease. Based on the Delphi methodology involving a multidisciplinary panel, 6 main dimensions of QoL were identified with high consensus: general health, physical health, mental health, daily functioning, social relationships, and economic and employment aspects. Furthermore, the inclusion of new underrepresented indicators such as dysautonomia and affective-sexual life was agreed upon, and the conceptual and linguistic precision of the items was improved through expert consensus.

Existing chronic illness literature highlights the importance of multiple life domains for understanding patients’ experiences [38–40]. In LC, quality of life is typically assessed with generic tools (EQ-5D-5 L, WHOQOL-BREF, SF-36) that cover broad physical, emotional, and social dimensions [6, 16, 41, 42]. However, these instruments miss many LC-specific impacts, including cognitive impairment, dysautonomia, affective-sexual life, work and financial consequences, and common symptoms like dyspnea [43–45]. Thus, while feasible and sensitive, current measures provide only a partial view of LC-related quality of life [46]. In contrast, the results of this study contribute to addressing these gaps by adding often-overlooked indicators, such as cognitive difficulties, dysautonomia symptoms, emotional and mental fatigue, reduced personal autonomy, affective-sexual life, perceived social support, social isolation, income loss, and return-to-work difficulties. Experts strongly supported including dysautonomia, affective-sexual life, and social support (consensus > 88%), underscoring their clinical and experiential importance. The findings align with previous research indicating that disease-specific tools tend to be more sensitive to clinical change [47–49].

Beyond generic tools, several condition-specific PROMs have been developed to characterize LC, primarily focusing on functional status (e.g., Post-COVID Functional Status Scale - PCFS [50]), symptom burden (e.g., Symptom Burden Questionnaire - SBQ™–LC [51]), and integrated symptom and disability assessment (e.g., COVID-19 Yorkshire Rehabilitation Scale - C19-YRS [52]). While these instruments are valuable for clinical characterization – the SBQ‑LC assesses symptom burden and the C19‑YRS functional rehabilitation status – they are not designed as multidimensional quality of life measures. In this landscape, specific LC tools such as the PAC-19QoL represent an important step forward in measuring the impact of this condition, as they capture a wide range of symptoms and life domains—including emotional well-being, cognitive difficulties, social relationships, and sexual interest [18]. Their extensive scope is a strength, offering a detailed clinical picture and strong content coverage. However, its comprehensive nature (44 items) may pose challenges in clinical settings where patient time and energy are limited, especially given common LC symptoms like chronic fatigue and brain fog.

In this context, the LC-6D-QoL Scale is proposed as a concise 16-indicator alternative organized into six conceptually defined dimensions, developed through qualitative interviews and refined by a panel of experts. While more concise, it still incorporates domains often underrepresented in generic measures, including dysautonomia [19, 53], psychosocial and sexual difficulties [54] and socioeconomic aspects. Its brevity aims to enhance usability in settings where patient time and energy are limited, without sacrificing coverage of domains identified as clinically and experientially relevant.

The LC-6D-QoL scale is clinically useful due to its brief format, which makes it practical for routine use and increases response rates, especially among patients with fatigue or cognitive issues. Its development involved patients from the outset, following PROM development standards for patient engagement and expert consensus [24]. The prior qualitative study [21] was exploratory and did not include formal cognitive debriefing; instead, the Delphi process provided content validity evidence through expert consensus, with patient‑experts assessing item clarity and relevance. Furthermore, the participation of four patient-experts enriched the consensus through their experiential perspective, aligning with evidence on patient engagement [55]. It thus provides a viable, patient-centered alternative, particularly useful in contexts with time constraints or longitudinal follow-ups.

The LC-6D-QoL is intended for screening (identifying affected domains) and monitoring (tracking changes over time) but should not be used as a confirmatory outcome measure (e.g., primary endpoint in clinical trials) until full psychometric validation is completed. For example, the scale could help identify areas where patients experience greatest limitations (e.g., physical function, mental health, daily activities). In clinical settings, this information could support referrals to appropriate services (e.g., physiotherapy for mobility issues, occupational therapy for daily living difficulties, mental health support for emotional distress, or autonomic function assessment for dysautonomia). Once the scale is psychometrically validated and minimal important differences are established, specific score thresholds might further guide these clinical decisions.

The present study reached consensus levels above 75% on all dimensions after the second round, which is in line with thresholds commonly used in the Delphi literature [34, 56]. In the first round, Kendall’s coefficient was W = 0.63 (*p* < 0.001), reflecting substantial agreement. In the second round, while greater homogeneity in ratings was achieved (IQR = 1.00 in all dimensions except one, which had an IQR = 0.00), the agreement on the prioritization of indicators was low (Kendall W = 0.22). This phenomenon has been documented in other Delphi studies with heterogeneous panels, where professional or epistemic differences affect ranking, although not necessarily inclusion [57, 58]; Kendall’s W may underestimate agreement in such contexts, and low values do not necessarily indicate a lack of consensus. Since the primary objective of the study—establishing the expert-informed foundation for future validation—was achieved with high consensus on the inclusion of all dimensions and indicators, the decision was made to conclude the process after the second round. A third round was considered unnecessary to advance this foundational aim and posed a risk of panelist fatigue without substantial benefit to the study’s core goal [34]. The low prioritization agreement (Kendall’s W = 0.22) reflects LC’s heterogeneity, supporting the scale’s use as a profiling tool where all dimensions carry individual clinical relevance.

The LC‑6D‑QoL is conceptualized as a profile measure, not a unidimensional construct. Its six dimensions are interrelated but not hierarchical, supporting dimension‑level interpretation; the total score awaits psychometric validation. The Delphi panel treated these dimensions as conceptually distinct; future factor analysis or IRT will empirically assess their independence. This consensus-based process thus positions the LC-6D-QoL Scale for subsequent psychometric testing. Further studies should now assess the scale’s psychometric properties, including reliability, validity, and cross-cultural adaptability. The LC-6D-QoL is conceptually designed to yield a total summary score (sum of 16 items, each scored 1–5; range 16–80, with higher scores indicating better quality of life). Therefore, at this stage, while the total score is conceptually proposed, its use in clinical or research settings should be considered exploratory pending full psychometric validation. Future psychometric validation—including factor analysis, internal consistency, responsiveness testing, and the minimal important difference—will determine whether a total score can be legitimately used.

Among the strengths of this study are the participation of experts from diverse disciplines and the use of qualitative and quantitative feedback between rounds, which allowed for collaborative construction of the instrument, anchored in evidence and clinical experience. Furthermore, the initial patient‑centered dimensions and indicators were derived from a prior qualitative study with 23 non‑professional Long COVID patients [21].

However, there are also some limitations. The 30% attrition rate is within the range commonly reported in Delphi studies [34] and likely reflects the time commitment required. Nevertheless, attrition may have influenced agreement metrics, as dropouts could have held differing views. The reduction in the number of experts in the second round may also have affected the stability of some metrics. In addition, the study was conducted in a predominantly Spanish cultural and healthcare context, with one participant from Mexico. Geographical concentration in these studies might restrict the applicability of the results to different populations, especially those with diverse healthcare systems, socio-economic contexts, or cultural backgrounds. The most context‑sensitive domains are Economic and occupational aspects (income loss, work adaptations) and Social relationships (perceived support), as these vary with healthcare systems, disability policies, and cultural norms.

Although responses were anonymized and only group summaries were presented, the Delphi method may introduce biases such as artificial convergence driven by controlled feedback, anchoring on aggregated results, or social desirability. These cannot be fully excluded. The four patient‑experts in the Delphi panel were also healthcare professionals, which may limit representativeness of the general LC population and underrepresent vulnerable groups.

The 5‑point Likert response format may be prone to floor or ceiling effects, particularly in patients with very mild or very severe LC. To assess this possibility, validation studies should examine response distributions and consider item response theory analyses. Also, single‑item indicators for complex constructs (e.g., mental health, social support) are brief and feasible but lack measurement depth; their reliability requires empirical confirmation. The one‑month recall period may not fully capture symptom variability in relapsing‑remitting Long COVID; future studies could compare this with shorter recall periods. Cognitive debriefing with a broader, non‑professional patient sample would further strengthen content validity. Future research should assess internal consistency, construct validity, responsiveness, the minimal important difference, and test-retest reliability in diverse patient samples and diverse socio‑demographic backgrounds.

## Conclusion

In conclusion, this study highlights the importance of a specific scale to assess quality of life in patients with Long COVID. The LC-6D-QoL Scale, based on patients’ experiences and developed through expert consensus using the Delphi methodology, captures six key dimensions, providing a more comprehensive and patient-centered assessment. By including indicators that are rarely explored in generic instruments—such as dysautonomia, cognitive difficulties, affective and sexual life, mental fatigue, and work-related impact—the scale addresses areas often overlooked in existing measures. Its brevity (16 indicators, each measured with a single item) may facilitate routine use in both clinical and research settings, including those with limited resources. These findings provide an expert-informed foundation for future validation of this multidimensional, patient-reported instrument that may better capture the complexity of Long COVID and help identify needs that remain under-recognized in healthcare systems.

## Electronic supplementary material

Below is the link to the electronic supplementary material.

Supplementary Material 1

## Data Availability

The datasets generated and/or analyzed during the current study are not publicly available due to the sensitive and identifiable nature of the qualitative data but are available from the corresponding author on reasonable request.
