# Supplementary material for: Development of the long COVID – 6 dimensions quality of life (LC-6D-QoL) scale: a Delphi study
Source: J Patient Rep Outcomes. 2026 May 20;10:128. doi: 10.1186/s41687-026-01084-3 (PMC13421521; doi:10.1186/s41687-026-01084-3)
Supplement: Supplementary file 1 — Supplementary Material 1 [file 41687_2026_1084_MOESM1_ESM.pdf]

Development of the Long COVID – 6 Dimensions Quality of Life (LC-6D-QoL) Scale: A Delphi Study.

Supplementary Information: Summary table of Delphi consensus by indicator

Dimension	Indicator	Consensus (%)	SD	Observation	First version	Final version
General Health	General health	84.62	0.87	No changes	How would you rate your overall health status?	
Physical Health	Pain	84.62	0.87	Reformulated for clarity	In the last month, how much has pain interfered with your daily life (including work and household tasks)?	In the last month, how much pain has interfered with your daily life (including work and household tasks)?
	Physical fatigue	88.46	0.80	No changes	In the last month, to what extent have you felt physically fatigued while performing everyday activities?	
	Functionality	84.62	0.87	No changes	In the last month, to what extent did you feel limited when making moderate effort, carrying groceries, climbing stairs, or walking for an hour?	
	Dysautonomia	92.31	0.86	New indicator added after Round 1		In the last month, have you experienced symptoms such as dizziness, rapid heartbeat, or excessive sweating when changing position (e.g., standing)?
Mental Health	Emotional symptoms	84.62	0.87	Expanded to include depression and anxiety	In the last month, to what extent have you felt depressed?	In the last month, to what extent have you felt depressed or anxious?
	Mental fatigue	84.62	0.87	No changes	In the last month, to what extent have you felt mentally fatigued while performing everyday activities?	
	Cognitive problems	84.62	0.87	No changes	In the last month, have you had difficulties concentrating or remembering essential information (e.g., appointments, names, or tasks)?	

Daily Functioning	Daily activity limitations	84.62	0.87	No changes	In the last month, to what extent have you felt limited in performing daily tasks, participating in leisure activities, or moving freely?	
	Personal autonomy	88.46	0.87	No changes	In the last month, how would you describe your level of autonomy in performing daily activities (personal hygiene, medication management, cooking)?	
Social Relationships and Support	Social support perception	92.31	0.74	Reformulated to emphasize understanding/ support	In the last month, how much have your relationships with family, friends or partner contributed to your physical and emotional well-being?	In the last month, to what extent did you feel your family and friends understand your situation and support you?
	Social isolation	84.62	0.87	No changes	In the last month, how often have you had contact with friends?	
	Affective and sexual life	88.46	0.70	New indicator added after Round 1		In the last month, how much has your health affected your romantic or sexual life?
Economic and Work-related Aspects	Income impact	80.77	0.83	No changes	In the last month, how much has your health affected your income?	
	Work/ academic performance	84.62	0.87	Reformulated from “job instability”	In the last month, how has your health affected your job stability (e.g., reduced working hours, contract changes)?	In the last month, to what extent has your health affected your work or academic performance?
	Return-to-work challenges	84.62	0.87	No changes	In the last month, have you needed adaptations at work (flexible hours, team support, or task adjustment)?	
